# Complete genome sequence of *Pseudomonas alcaliphila* JAB1 (=DSM 26533), a versatile degrader of organic pollutants

**DOI:** 10.1186/s40793-017-0306-7

**Published:** 2018-02-01

**Authors:** Jakub Ridl, Jachym Suman, Serena Fraraccio, Miluse Hradilova, Michal Strejcek, Tomas Cajthaml, Andrea Zubrova, Tomas Macek, Hynek Strnad, Ondrej Uhlik

**Affiliations:** 1 Department of Genomics and Bioinformatics, Institute of Molecular Genetics, Academy of Sciences of the Czech Republic, Prague, Czech Republic; 2 Department of Biochemistry and Microbiology, Faculty of Food and Biochemical Technology, University of Chemistry and Technology, Prague, Czech Republic; 3 Laboratory of Environmental Biotechnology, Institute of Microbiology, Academy of Sciences of the Czech Republic, Prague, Czech Republic

**Keywords:** *Pseudomonas alcaliphila* JAB1, *Pseudomonadaceae*, Genome, Dioxygenase, Monooxygenase, Biodegradation, Bioremediation, Aromatic compounds, Biphenyl, Polychlorinated biphenyls (PCBs), Chlorobenzoic acids (CBAs), *cis*-1,2-dichloroethylene (cDCE), Phenol, *bph* genes, *ben* genes, *phe* genes, MALDI-TOF MS

## Abstract

In this study, following its isolation from contaminated soil, the genomic sequence of *Pseudomonas alcaliphila* strain JAB1 (=DSM 26533), a biphenyl-degrading bacterium, is reported and analyzed in relation to its extensive degradative capabilities. The *P. alcaliphila* JAB1 genome (GenBank accession no. CP016162) consists of a single 5.34 Mbp-long chromosome with a GC content of 62.5%. Gene function was assigned to 3816 of the 4908 predicted genes. The genome harbors a *bph* gene cluster, permitting degradation of biphenyl and many congeners of polychlorinated biphenyls (PCBs), a *ben* gene cluster, enabling benzoate and its derivatives to be degraded, and *phe* gene cluster, which permits phenol degradation. In addition, *P. alcaliphila* JAB1 is capable of cometabolically degrading *cis*-1,2-dichloroethylene (cDCE) when grown on phenol. The strain carries both catechol and protocatechuate branches of the β-ketoadipate pathway, which is used to funnel the pollutants to the central metabolism. Furthermore, we propose that clustering of MALDI-TOF MS spectra with closest phylogenetic relatives should be used when taxonomically classifying the isolated bacterium; this, together with 16S rRNA gene sequence and chemotaxonomic data analyses, enables more precise identification of the culture at the species level.

**Electronic supplementary material:**

The online version of this article (10.1186/s40793-017-0306-7) contains supplementary material, which is available to authorized users.

## Introduction

Over recent decades, significant quantities of potentially harmful chemicals have been released into the environment, creating countless numbers of contaminated sites. Major contaminants include halogenated and nitrated alicyclic, aliphatic, aromatic and polyaromatic compounds of industrial and agricultural origin. Many of these compounds have been found to have toxic, mutagenic and carcinogenic effects on living organisms. Removal of these xenobiotics usually involves physical and chemical processes, such as landfill, excavation and incineration, which are expensive and difficult to execute. An alternative approach, bioremediation, uses ubiquitous plant-microbe interactions to degrade xenobiotics [1]. Bacteria and fungi are natural recyclers capable of funneling toxic organic compounds to central metabolism intermediates [2], thereby creating harmless products [3] such as carbon dioxide and water. In addition to the enormous phylogenetic diversity of microorganisms, the richness of their metabolic activities promotes the degradation of pollutants and xenobiotics in different environments.

Members of the genus *Pseudomonas* [4, 5]*,* one of the most diverse bacterial genera, inhabit several environmental niches and have been studied in relation to human and plant pathogenicity, antibiotic resistance, plant growth promotion, plant-derived organic matter degradation and bioremediation [6]. Pseudomonads, which are metabolically highly versatile, contain both abundant and unique metabolic pathways [7], which, most importantly, catabolize a broad range of substrates. Many of these substrates are pollutants, including aliphatic and aromatic petroleum hydrocarbons [8–12], BTEX [13, 14], phenolic compounds ranging from phenol via methylphenols and nitrophenols to chlorophenols [15], benzoate, CBAs and toluates [16], biphenyl and PCBs [17–19], chlorinated aliphatics [20] and many others. In this study, we present the first complete genome of the species *P. alcaliphila*, strain JAB1, whose extensive biodegradation capabilities are highlighted.

## Organism information

### Classification and features

**Fig. 1:**
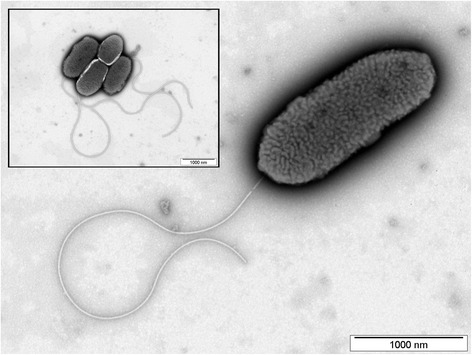
Transmission electron photomicrograph of *P. alcaliphila* JAB1

*P. alcaliphila* was described as a facultatively psychrophilic alkaliphilic species isolated from seawater off the coast of Rumoi, Hokkaido, Japan. The characteristics of this species are as follows: alkaliphile, incapable of growth at >40 °C, catalase- and oxidase-positive and also capable of reducing nitrate to nitrite and of hydrolyzing casein and gelatin [21]. Further physiological features are listed in Table 1. The JAB1 cells are monotrichous rods as shown in Fig. 1.

**Table 1:** Classification and general features of *P. alcaliphila* JAB1

MIGS ID	Property	Term	Evidence code^a^
	Classification	Domain *Bacteria*	TAS [55]
		Phylum *Proteobacteria*	TAS [56, 57]
		Class *Gammaproteobacteria*	TAS [58, 59]
		Order *Pseudomonadales*	TAS [5, 60]
		Family *Pseudomonadaceae*	TAS [61]
		Genus *Pseudomonas*	TAS [4, 5]
		Species *Pseudomonas alcaliphila*	TAS [21]
		Strain JAB1 (Accession no. DSM 26533)	
	Gram stain	Negative	TAS [21]
	Cell shape	Rod-shaped	IDA, TAS [21]
	Motility	Motile	IDA, TAS [21]
	Sporulation	Non-sporulating	TAS [21]
	Temperature range	Mesophile	IDA
	Optimum temperature	28–37 °C	IDA
	pH range; Optimum	Not tested; Neutral	TAS [21]
	Carbon source	Biphenyl, phenol, other organic substrates	IDA
MIGS-6	Habitat	Soil	TAS [22]
MIGS-6.3	Salinity	Up to 7% NaCl (*w*/*v*)	TAS [21]
MIGS-22	Oxygen requirement	Aerobic	TAS [22]
MIGS-15	Biotic relationship	Free-living	TAS [22]
MIGS-14	Pathogenicity	Non-pathogen	NAS
MIGS-4	Geographic location	Czech Republic	TAS [22]
MIGS-5	Sample collection	2000	NAS
MIGS-4.1	Latitude	50°1′52″N	NAS
MIGS-4.2	Longitude	16°35′55″E	NAS
MIGS-4.4	Altitude	420 m	NAS

*IDA* Inferred from Direct Assay, *TAS* Traceable Author Statement (i.e., a direct report exists in the literature), *NAS* Non-traceable Author Statement (i.e., not directly observed for the living, isolated sample, but based on a generally accepted property for the species, or anecdotal evidence)

^a^Evidence codes

**Fig. 2:**
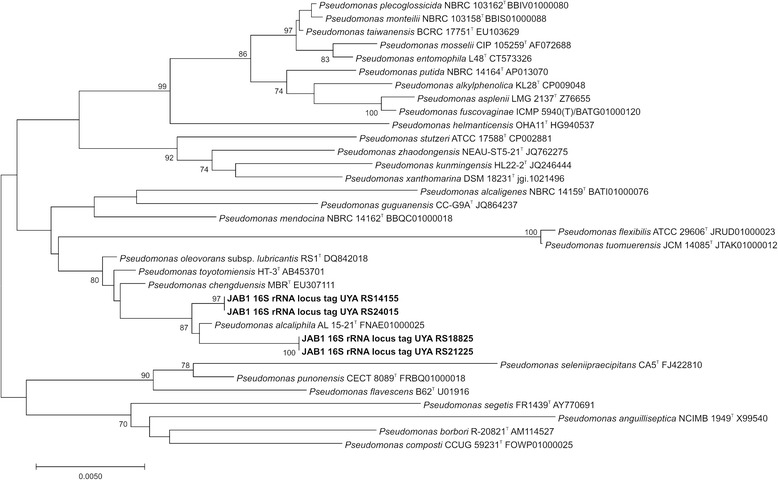
Phylogenetic tree indicating current phylogenetic placement of strain JAB1.The phylogenetic tree was constructed in MEGA7 [62] using secondary structure-based alignment of 16S rRNA gene sequences of the JAB1 strain (four copies of the 16S rRNA gene retrieved from the JAB1 genome) and its closest relatives [63]. The evolutionary distances were computed using the Kimura 2-parameter method [64]. All positions with less than 95% site coverage were eliminated; there were a total of 1375 positions in the final dataset. The tree construction method used was Neighbor-Joining [65]. The bootstrap test (1000 replicates) was used to test tree topology [66]; only bootstrap values >70 are indicated

**Fig. 3:**
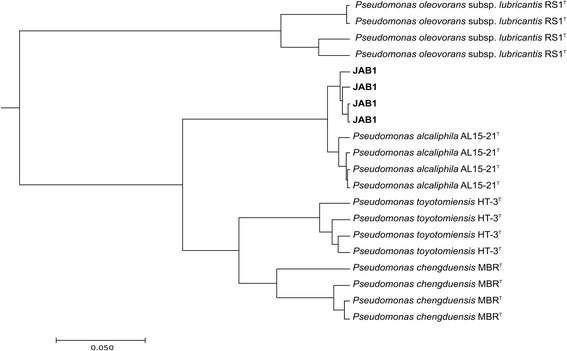
Clustering of MS spectra of strain JAB1 and related pseudomonads

The JAB1 strain was originally misidentified as *P. pseudoalcaligenes* [22]. The consensus 16S rRNA gene sequence, compiled from four 16S rRNA gene copies contained in the JAB1 genome, had 99.93% similarity to those of *P. alcaliphila* AL 15-21^T^ [21], *P. chengduensis* MBR^T^ [23] and *P. oleovorans* subsp. *lubricantis* RS1^T^ [24]. Additional closest matches included *P. toyotomiensis* HT-3^T^ (99.86% similarity) and *P. mendocina* CH50^T^ (99.24% similarity). A phylogenetic tree indicates closest relatedness of the JAB1 strain to *P. alcaliphila* AL 15-21^T^ (Fig. 2). In addition, the JAB1 strain was unable to grow at 41 °C, which is a typical feature of *P. alcaliphila* but not of other closely related pseudomonads [23]. Furthermore, whole-cell MALDI-TOF MS analysis, performed following the methodology described elsewhere [12], indicated that JAB1 spectra clustered with those of *P. alcaliphila* AL 15-21^T^ (Fig. 3). The results of MALDI-TOF MS profiling thus further confirmed the identity of the JAB1 strain as *P. alcaliphila*. Therefore, we propose that MALDI-TOF MS analysis be performed of the isolate and its closest phylogenetic relatives when taxonomically classifying the isolated bacterium. In addition to 16S rRNA gene sequence and chemotaxonomic data analysis, MALDI-TOF MS can provide additional information resulting in more precise classification of the isolate.

### Extended feature descriptions: biodegradative capabilities

The JAB1 strain was isolated from legacy contaminated soil in Jablonné nad Orlicí in the Czech Republic as a biphenyl-degrading bacterium capable of extensive degradation of several congeners of PCBs when grown in the presence of biphenyl [22]. PCB-degradation capabilities were further determined by a resting cell assay. Briefly, microcosms (volume of 119 ml) bearing biphenyl-preinduced *P. alcaliphila* JAB1 mid-log phase culture resuspended in mineral salt solution (20 ml per microcosm) were spiked with a 0.001% (*w*/*v*) commercial mixture of PCBs Delor 103 and incubated for 48 h. The content of individual PCB congeners present in the microcosms as well as CBA accumulation were determined using GC-MS (450-GC, 240-MS ion trap detector, Varian, Walnut Creek, CA). PCBs were analyzed in ethyl acetate extracts according to the method described by Čvančarová M. et al. [25]. CBAs were analyzed in the extracts using GC-MS after methylation with diazomethane according to our previously published protocol [26]. The respective chemical standards for the analytes were obtained from Merck (Darmstadt, Germany), Supelco (Steinheim, Germany), TCI Europe (Zwijndrecht, Belgium) and AccuStandard (New Haven, USA).

**Fig. 4:**
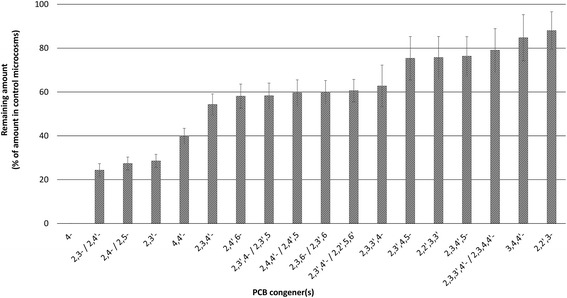
Degradation activities of strain JAB1 towards PCB congeners. Biphenyl-induced JAB1 cells were co-incubated with commercial PCB mixture Delor 103 for 48 h, with individual congener depletion being determined by GC-MS. Degradation of the following biphenyl derivatives was monitored but not observed: 2,2′-diCl, 2,2′,3-triCl, 2,2′,5-triCl, 2,2′,4-triCl, 2,2′,4,6′-tetraCl, 2,2′,3,6-tetraCl, 2,2′,3,6′-tetraCl, 2,2′,5,5′-tetraCl, 2,2′,4,5′-tetraCl, 2,2′,4,5 tetraCl, 2,2′,4,4′-tetraCl, 2,2′,3,5′-tetraCl, 2,2′,3,4′-tetraCl, 2,3′,4′,6-tetraCl, 2,2′,3,4 tetraCl, 2,3,4′,6-tetraCl, 2,4,4′,5-tetraCl, 2,3′,4′,5-tetraCl, 2,3′,4′,5′-tetraCl, 2,3′,4,4′-tetraCl, 3,3′,4,4′-tetraCl, 2,2′,3,4′,6-pentaCl, 2,2′,3,4,6′-pentaCl, 2,2′,4,5,5′-pentaCl, 2,3,3′,5′,6-pentaCl, 2,2′,4,4′,5 pentaCl, 2,2′,3,4′,5′-pentaCl, 2,2′,3,4,5′-pentaCl, 2,3,3′5,5′-pentaCl, 2,3,3′,4′,6-pentaCl, 2,2′,3,3′,4-pentaCl, 2,3′,4,4′,5-pentaCl, 2,3,3′,4,4′-pentaCl

Metabolic activity of the JAB1 strain resulted in the depletion of various mono-, di-, tri- and tetra-chlorinated biphenyls as shown in Fig. 4. At the same time, 2-CBA, 3-CBA, 4-CBA, 2,3-diCBA, 2,4-diCBA and 2,5-diCBA formation (data not shown) was observed over the course of the coincubation period; these are common biodegradation intermediates of various PCB congeners and products of the upper PCB degradation pathway.

The degradation of cDCE was assessed during growth on phenol, a compound known to promote cDCE degradation in microbial cultures [27]. Microcosms (volume of 119 ml) bearing *P. alcaliphila* JAB1 culture (20 ml per microcosm) grown and repeatedly propagated on 1 mM phenol and 0.01 mM cDCE were monitored for phenol and cDCE depletion as described elsewhere [28] as well as the increase in microbial culture density measured spectrophotometrically. Additional cultures were prepared in the presence of 0.01 mM cDCE as sole carbon source and of 0.01 mM cDCE and 1 mM sodium pyruvate to exclude direct utilization of cDCE by JAB1. In addition, SPME-GC-MS analysis of culture headspaces was performed over time in order to identify possible volatile chlorinated intermediates of the cDCE degradation pathway.

**Fig. 5:**
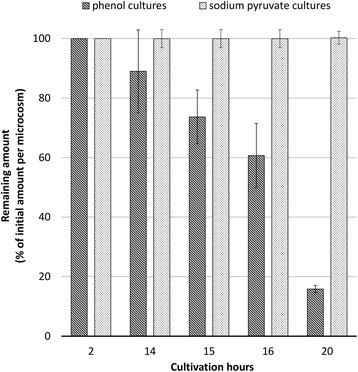
Trend in cDCE depletion over time in JAB1 cultures grown on 1 mM phenol or 1 mM sodium pyruvate in the presence of 0.01 mM cDCE. Residual amounts of cDCE over time are expressed as a percentage of initial cDCE content. Error bars indicate the standard deviation among three biological replicates

Monitoring of cDCE levels in cultures grown on 1 mM phenol revealed consistent cDCE depletion after the first 14 h of cultivation, when phenol (the growth-supporting substrate) was completely or almost completely consumed (Fig. 5). After 72 h of cultivation, 100% cDCE depletion was attained exclusively in phenol-grown cultures; during the same time period, cDCE loss accounted for only 13.4% ± 8.8 pp. in sodium pyruvate-grown cultures, 9.7% ± 9.1 pp. for JAB1 cultures exposed solely to cDCE and 12.5% ± 1.2 pp. in sterile medium. Such minimal cDCE loss under conditions tested can be ascribed to non-biological processes. Proven total cDCE depletion only in phenol-grown cultures confirms our hypothesis that cDCE degradation occurs cometabolically. To date, 1,1-dichloroacetaldehyde was confirmed as a degradation intermediate by means of SPM-GC-MS in culture headspaces. Quantification of this molecule and its accumulation over time will be an area for future research together with the study of possible metabolic pathways to promote further transformation of 1,1-dichloroacetaldehyde.

## Genome sequencing information

### Genome project history

The JAB1 strain was selected for sequencing due to its extensive degradative capabilities. The genome project was initiated in 2010. The complete genome sequence, deposited in GenBank under the accession number CP016162, was released public on April 14, 2017. A summary of the project information and its association with MIGS standard is shown in the Table 2.

**Table 2:** Genome sequencing project information for *P. alcaliphila* JAB1

MIGS ID	Property	Term
MIGS 31	Finishing quality	Finished
MIGS-28	Libraries used	454 shotgun, 454 8 kb paired-end
MIGS 29	Sequencing platforms	GS FLX+
MIGS 31.2	Fold coverage	41.5
MIGS 30	Assemblers	Newbler 2.8
MIGS 32	Gene calling method	GeneMarkS+
	Locus Tag	UYA
	Genbank ID	CP016162
	GenBank Date of Release	April 14, 2017
	GOLD ID	Gp0021677
	BIOPROJECT	PRJNA104953
MIGS 13	Source Material Identifier	DSM 26533
	Project relevance	Bioremediation, aromatic compounds degradation

### Growth conditions and genomic DNA preparation

Following its isolation from soil, the culture was preserved in a mineral salt solution [12] with biphenyl as sole carbon source and, over the long term, in glycerol stocks prepared from growing liquid cultures. For the purposes of DNA isolation, the culture was grown overnight on plate count agar (Difco, UK) at 28 °C. Genomic DNA was isolated using the PureLink™ Genomic DNA Mini Kit (Invitrogen, USA) according to the manufacturer’s instructions.

### Genome sequencing and assembly

The genomic DNA of *P. alcaliphila* JAB1 was used to prepare shotgun and 8 kb paired-end 454 sequencing libraries according to the Library Preparation Method Manual (Roche). These libraries were sequenced with the GS FLX instrument using GS FLX+ chemistry (Roche) at the Institute of Molecular Genetics AS CR (Prague, Czech Republic). The resulting 110,702 shotgun and 461,976 paired-end reads comprised a total of 221,447,771 bases and represented 41.5-fold genome coverage. These reads were assembled in Newbler 2.8 software (Roche), and gaps were closed by local assembly of selected reads in Staden software [29] into a single circular contig.

### Genome annotation

The NCBI Prokaryotic Genome Annotation Pipeline [30] was used for automated genome annotation, and functional annotations were carried out by searching against KEGG [31], COG [32] and Pfam [33] databases. SignalP [34] and TMHMM [35] tools were used for the prediction of genes with signal peptides and transmembrane helices, respectively. Searches against NCBI-NR [36], RefSeq [37], UniProtKB/Swiss-prot [38] and TCDB [39] were carried out for functional assignment of selected genes.

## Genome properties

**Fig. 6:**
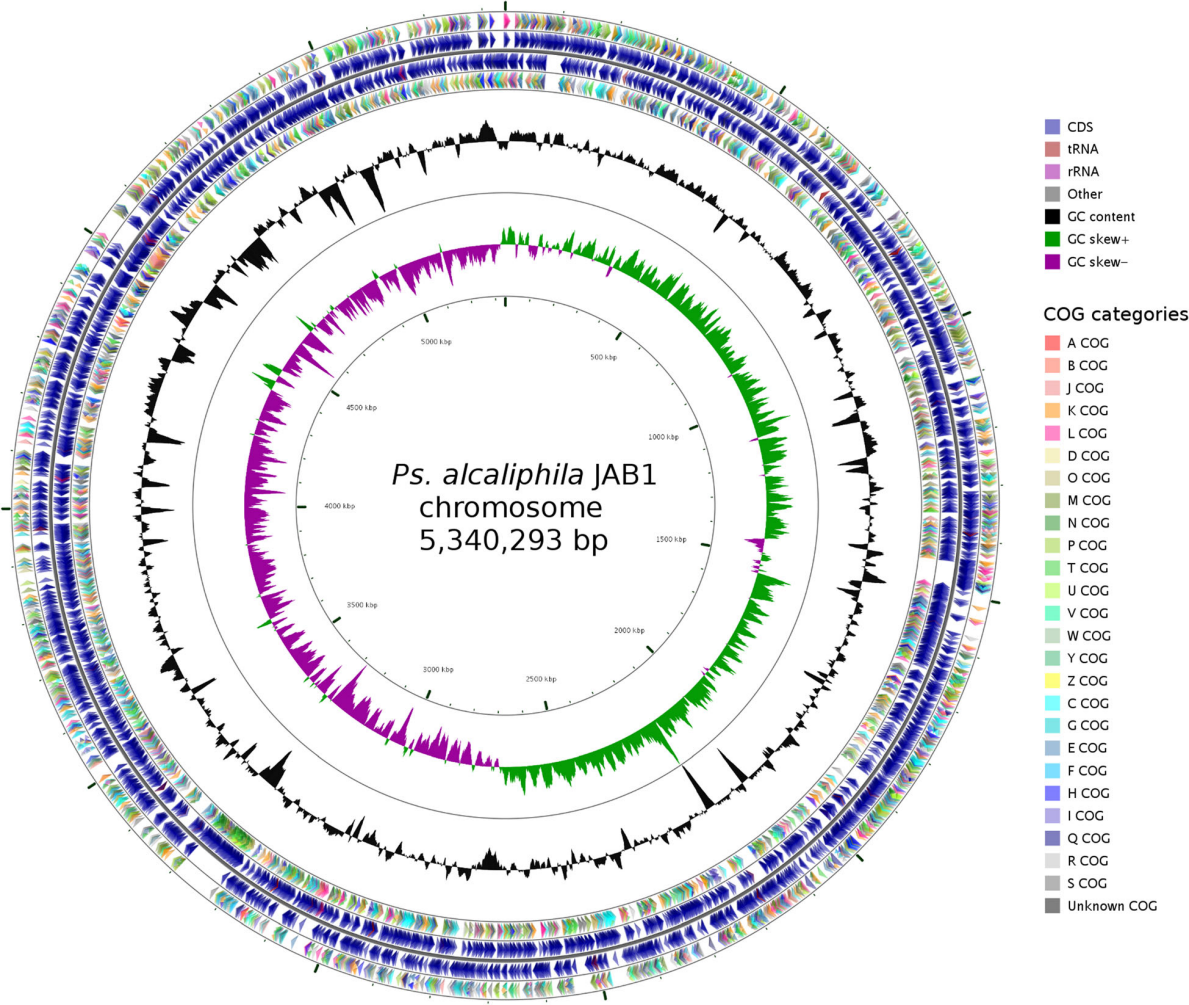
Circular map of the *P. alcaliphila* JAB1 chromosome. From outside to the center: CDS on forward strand colored according to their COG categories, all CDS and RNA genes on forward strand, all CDS and RNA genes on reverse strand, CDS on reverse strand colored according to their COG categories, GC content, and GC skew. The map was generated using CGView [67]

The *P. alcaliphila* JAB1 genome consists of a single 5,340,293 bp-long chromosome with a GC content of 62.5% (Fig. 6). The 4908 predicted genes correspond to an 89% coding density. The genome contains 4773 CDS, 65 tRNA genes for all 20 amino acids, 4 rRNA operons, 4 ncRNA genes, 3 CRISPR repeats and 54 pseudogenes. Function was assigned to 3816 CDS, with 957 CDS annotated as hypothetical proteins only (Table 3). The distribution of COG functional categories is shown in Table 4.

**Table 3:** Genome statistics

Attribute	Value	% of Total
Genome size (bp)	5,340,293	100
DNA coding (bp)	4,749,316	88.93
DNA G + C (bp)	3,339,724	62.54
DNA scaffolds	1	
Total genes	4908	100
Protein coding genes	4773	97.25
RNA genes	81	1.65
Pseudo genes	54	1.10
Genes in internal clusters	NA	
Genes with function prediction	3816	77.75
Genes assigned to COGs	3927	80.01
Genes with Pfam domains	4221	86.00
Genes with signal peptides	557	11.35
Genes with transmembrane helices	1139	23.21
CRISPR repeats	3	0.06

**Table 4:** Number of genes associated with general COG functional categories

Code	Value	%age	Description
J	189	3.96	Translation, ribosomal structure and biogenesis
A	1	0.02	RNA processing and modification
K	368	7.71	Transcription
L	216	4.53	Replication, recombination and repair
B	2	0.04	Chromatin structure and dynamics
D	43	0.90	Cell cycle control, Cell division, chromosome partitioning
V	59	1.24	Defense mechanisms
T	425	8.90	Signal transduction mechanisms
M	232	4.86	Cell wall/membrane biogenesis
N	133	2.79	Cell motility
U	107	2.24	Intracellular trafficking and secretion
O	176	3.69	Posttranslational modification, protein turnover, chaperones
C	285	5.97	Energy production and conversion
G	196	4.11	Carbohydrate transport and metabolism
E	433	9.07	Amino acid transport and metabolism
F	88	1.84	Nucleotide transport and metabolism
H	179	3.75	Coenzyme transport and metabolism
I	189	3.96	Lipid transport and metabolism
P	256	5.36	Inorganic ion transport and metabolism
Q	118	2.47	Secondary metabolites biosynthesis, transport and catabolism
R	574	12.03	General function prediction only
S	423	8.86	Function unknown
–	846	17.72	Not in COGs

The total is based on the total number of protein coding genes in the genome

## Insights from the genome sequence

The genome harbors approximately 50 oxygenase genes, many of which are relevant to the biodegradation capabilities of the strain, especially those encoding for mono- and di-oxygenases responsible for the hydroxylation and opening of the aromatic ring, which are crucial steps in aromatic xenobiotic degradation (see below for details). Several other genes found are associated with the heavy metal resistance of the strain (e.g. heavy metal efflux P-type ATPases), indicating its overall adaptation to contaminated environments; organic compound contamination is often accompanied by high concentrations of heavy metals [40, 41]. The genome contains a complete set of genes for flagellar assembly and 50 additional genes associated with chemotaxis. Roughly 70 of the predicted CDS are putative intact or mutated transposase genes and phage-related genes.

### Extended insights: degradation of aromatic compounds

In total, five regions harboring genes for aromatic compound degradation pathways were identified in the JAB1 genome as shown in Additional file 1: Table S1.

The first region, position range 1,474,745–1,486,552 in the annotated genome sequence, harbors genes encoding for a three-component benzoate 1,2-dioxygenase (*benABC* genes) followed by enzymes of a pathway enabling the complete degradation of benzoates and benzoate derivatives, yielding acetyl-CoA and pyruvate (catechol *meta*-cleavage pathway encoded by *benD*, *fdx*, and *dmp* cluster). The role played by the gene designated *nahX* encoding for putative ATP-cob(I)alamin adenosyltransferase in aromatic compound degradation is unclear [42]. No transcriptional regulator was found within or adjacent to this region, leading us to assume that genes from this region are not transcribed or their transcription is mediated *in trans* by regulatory proteins encoded elsewhere.

The second region, position range 1,518,873–1,540,279, harbors genes encoding for complete salicylate degradation via the catechol *meta*-cleavage pathway (*sal*/*fdx genes*), with salicylate 1-monooxygenase (encoded by *salA*) being responsible for opening the aromatic ring. Genes *antAB* encoding for large and small subunits of a putative terminal dioxygenase were also found in this region. Apart from genes presumed to be involved in the enzymatic degradation of aromatic compounds, ORFs encoding for transport proteins were found in this second region, namely a putative benzoate:H^+^ symporter (encoded by gene *benE*), a multi-component ABC transporter (six ORFs, designated *ORF1–6*) and a porin protein (*benK*), which are most likely involved in aromatic compound uptake. BenK ortholog has been shown to be involved in benzoate uptake in *Acinetobacter* sp. ADP1 [43]. Moreover, BenE and BenK orthologs from *P. putida* have been demonstrated to act as benzoate uptake proteins when heterologously expressed in yeasts [44]. Nevertheless, detailed experimental evidence is lacking on the function of these proteins and their role in aromatics/aromatic xenobiotics degradation, which represents a potential challenge for future research [45, 46].

The third region, position range 1,545,237–1,557,889, harbors *bph* genes encoding for enzymes of the complete upper and lower biphenyl degradation pathway. The structure of this operon is virtually identical to that of the *bph* operon found in the genome of the model PCB-degrading strain *P. pseudoalcaligenes* KF707 [47]. While pentadiene and its chlorinated derivatives can be degraded by enzymes encoded by the same operon (*bphHIJ* genes), no ORF encoding for benzoate terminal dioxygenase, essential for the completion of biphenyl degradation, is present in this region; nevertheless, such genes are found elsewhere in the JAB1 genome. Two other ORFs, *tbuX* and a gene designated *ORF7*, encoding for putative aromatic-transporter transmembrane proteins, were found in this region. Although the TbuX protein has been reported to be involved in the utilization of toluene in *Ralstonia pickettii* PKO1 [48], no detailed information exists on the precise role played by this group of proteins in aromatic compound transport. The predicted *ORF7*-encoded protein exhibits homology with members of the BphX family. Despite being commonly found in upper biphenyl degradation pathway-encoding supra-operonic clusters of various taxa, the function of these proteins in aromatic degradation is not known [49–51].

The fourth region, position range 4,397,470–4,411,693, harbors gene cluster *pheKLMNOP* encoding for multi-component phenol 2-monoxygenase and an *fdx* gene encoding a chloroplast-type ferredoxin essential for electron delivery to the active center of the oxygenase. The adjacent *dmp* cluster encodes for enzymes responsible for the *meta*-cleavage pathway of catechol/catechol derivatives, yielding acetyl-CoA and pyruvate. In this study, we demonstrate that strain JAB1 is capable of utilizing phenol as a sole carbon source and cometabolite of cDCE degradation.

The fifth region, position range 4,445,675 to 4,469,934, contains *benABCD* genes, which are responsible for the transformation of benzoate into catechol, as well as *catABC* and *pcaDIJF* genes, which encode the enzymes of the catechol branch of the β-ketoadipate pathway, yielding succinyl-CoA and acetyl-CoA [52]. This region also harbors *pcaHGB* genes for the protocatechuate branch of the β-ketoadipate pathway which enable hydroxybenzoate degradation; the *pobA* gene encoding 4-hydroxybenzoate 3-monooxygenase, responsible for the first step of 4-hydroxybenzoate hydroxylation, is located upstream of this fifth region (positions 4,435,851 to 4,437,041). In addition, this region harbors three ORFs encoding for putative transport proteins, which are most likely involved in aromatic compound uptake, designated as *ORF8* (encoding for outer membrane porin from the OprD family), *pcaK* (an ortholog of *p*-hydroxybenzoate and protocatechuate transport protein from the Major Facilitator Superfamily [53]) and *benE* (benzoate:H^+^ symporter from the BenE family). Two other ORFs encoding for a cell division protein (*zapE* gene) [54] and a permease of unknown substrate specificity and function (designated *ORF9*) are also present in the fifth region in a *pcaHG*-*zapE*-*ORF9* arrangement. As the intergenic region between the *pcaG* and *zapE* genes is only 1 bp in length, with *zapE* and *ORF9* sequences even overlapping by 5 bp, we hypothesize that *pcaHG*-*zapE*-*ORF9* represents a single transcriptional unit. The positioning of one or more genes involved in cell division as part of a cistron together with aromatic-degradation genes is rather unusual.

Since the regions harboring degradation-determining genes are flanked by transposase and conjugation protein-coding genes, and, at the same time, they generally exhibit analogous architecture and high similarity to corresponding orthologs from other pseudomonads, we hypothesize that their acquisition in the JAB1 genome was most likely due to multiple horizontal gene transfer events. However, further study is required to confirm this hypothesis.

## Conclusions

Thorough microbiological experiments and whole genome sequence analysis lead us to conclude that *P. alcaliphila* JAB1 is a versatile degrader of organic pollutants. Its extensive degradative capabilities are enabled by a variety of genes determining the degradation of both biphenyl/biphenyl derivatives (mediated by the biphenyl degradation pathway encoded by the *bph* cluster) and monocyclic aromatics such as benzoate and its derivatives (halobenzoates, toluates), salicylic acid and phenol/phenolic compounds.

## Additional file

Additional file 1: Table S1. Description of five regions harboring genes for aromatic compound degradation pathways were identified in the JAB1 genome. (DOCX 34 kb)

## Abbreviations

BTEX: Benzene, toluene, ethylbenzene, xylene
CBAs: Chlorobenzoic acids
cDCE: *cis*-1,2-dichloroethylene
diCBA: Dichlorobenzoic acid
GC-MS: Gas chromatography-mass spectrometry
MALDI-TOF MS: Matrix-assisted laser ionization/desorption-time of flight mass spectrometry
PCBs: Polychlorinated biphenyls
pp: Percentage points
