# Supplementary material for: Complete genome sequence of Pseudomonas alcaliphila JAB1 (=DSM 26533), a versatile degrader of organic pollutants
Source: Stand Genomic Sci. 2018 Feb 1;13:3. doi: 10.1186/s40793-017-0306-7 (PMC5796565; doi:10.1186/s40793-017-0306-7)
Supplement: Additional file 1: Table S1. — Description of five regions harboring genes for aromatic compound degradation pathways were identified in the JAB1 genome. (DOCX 34 kb) [file 40793_2017_306_MOESM1_ESM.docx]

| **Gene des­ignation** | **Coding sequence length [bp]** | **Position in the genome^*)^**  **5’-end:3’-end strand** | **GC content [%]** | **The closest BLASTP hit^**)^**  **[organism of origin]** | **Identity/ similarity [%]**^**)^ | **Protein length [aa]/ predicted molecular weight of protein [kDa]^***)^** | **Presumed activity**  **(EC number)** | **Catabolic pathway/function** | |
| --- | --- | --- | --- | --- | --- | --- | --- | --- | --- |
| ***benA*** | 1356 | 1474745:1476100  forward | 59 | benzoate 1,2-dioxygenase large subunit [*Ps. aeruginosa*] | 99/100 | 451/51.4 | Benzoate/toluate  1.2-dioxygenase large subunit  (EC 1.14.12.10) | Benzoate/benzoate-derivatives degradation (terminal dioxygenase) | |
| ***benB*** | 489 | 1476101:1476589  forward | 57 | toluate 1,2 dioxygenase subunit (plasmid) [*Ps. putida*] | 99/99 | 162/ 19.4 | Benzoate/toluate 1.2-dioxygenase small subunit  (EC 1.14.12.10) |  |  |
| ***benC*** | 1011 | 1476599:1477609 forward | 61 | toluate 1,2-dioxygenase electron transfer component  [*Ps. saponiphila*] | 99/99 | 336/36.3 | Benzoate/toluate 1.2-dioxygenase reductase subunit  (EC 1.14.12.10) |  |  |
| ***benD*** | 777 | 1477725:1478501 forward | 63 | 1,6-dihydroxycyclohexa-2,4-diene-1-carboxylate dehydrogenase  [*Ps. stutzeri*] | 98/99 | 258/27.9 | 1,6-dihydroxycyclohexa-2,4-diene-1-carboxylate dehydrogenase  (EC 1.3.1.25) | Benzoate/benzoate-derivatives degradation | |
| ***fdx*** | 333 | 1478646:1478978 forward | 64 | terredoxin  [*Ps. stutzeri*] | 96/97 | 110/11.7 | Ferredoxin, plant-type |  | |
| ***dmpB*** | 924 | 1478975:1479898 forward | 58 | catechol 2,3-dioxygenase  [*Pseudomonas* sp.] | 96/98 | 307/35.2 | Catechol 2,3-dioxygenase  (EC:1.13.11.2) | Catechol degradation (*meta* cleavage pathway) | |
| ***dmpC*** | 1461 | 1479933:1481393 forward | 66 | 2-hydroxymuconic semialdehyde dehydrogenase  [*Ps. aeruginosa*] | 99/99 | 486/51.6 | 2-hydroxymuconate-6-semialdehyde dehydrogenase  (EC: 1.2.1.85) |  |  |
| ***dmpD*** | 849 | 1481404:1482252 forward | 67 | 2-hydroxy-6-oxo-2,4-heptadienoate hydrolase  [*Ps. abietaniphila*] | 99/99 | 282/31.3 | 2-hydroxymuconic semialdehyde hydrolase  (EC 3.7.1.9) |  |  |
| ***dmpE*** | 786 | 1482264:1483049 forward | 65 | 2-oxopent-4-enoate hydratase  [*Ps. stutzeri*] | 99/100 | 261/27.9 | 2-oxopent-4-enoate hydratase  (EC:4.2.1.80) |  |  |
| ***dmpF*** | 939 | 1483064:1484002 forward | 61 | acetaldehyde dehydrogenase (acylating)  [*Ps. putida*] | 98/98 | 312/32.9 | acetaldehyde dehydrogenase (acylating)  (EC:1.2.1.10) |  |  |
| ***dmpG*** | 1038 | 1484013:1485050 forward | 63 | 4-hydroxy-2-oxovalerate aldolase  [*Ps. putida*] | 99/99 | 345/37.4 | 4-hydroxy 2-oxovalerate aldolase  (EC:4.1.3.39) |  |  |
| ***dmpH*** | 795 | 1485047:1485841 forward | 64 | 4-oxalocrotonate decarboxylase  [*Ps. stutzeri*] | 99/99 | 264/28.6 | 2-oxo-3-hexenedioate decarboxylase  (EC:4.1.1.77) |  |  |
| ***dmpI*** | 192 | 1485890:1486081 forward | 60 | 4-oxalocrotonate tautomerase  [*Ps. stutzeri*] | 98/100 | 63/6.8 | 4-oxalocrotonate tautomerase (EC:5.3.2.6) |  |  |
| ***nahX*** | 441 | 1486112:1486552 forward | 65 | heme-binding protein  [*Pseudomonas* spp.] | 100/100 | 146/15.5 | ATP-cob(I)alamin adenosyltransferase  (unknown function) | Unknown | |
|  |  |  |  |  |  |  |  |  | |
| ***benE*** | 1251 | 1518873:1520123  reverse | 60 | benzoate transport protein  [*Ps. pseudoalcaligenes* KF707] | 100/100 | 416/43.4 | benzoate:H^+^ Symporter (BenE) Family  (TCDB no. 2.A.46) | Benzoate/benzoate derivatives uptake | |
| ***antA*** | 1278 | 1520631:1521908  reverse | 61 | large terminal subunit of phenylpropionate dioxygenase  [*Pseudoxanthomonas spadix* BD-a59] | 97/98 | 425/48.0 | anthranilate 1,2-dioxygenase large subunit  (EC 1.14.12.1) | Aromatics degradation (terminal dioxygenase) | |
| ***antB*** | 465 | 1521949:1522413  reverse | 56 | anthranilate 1,2-dioxygenase small subunit  [*Pseudoxanthomonas spadix*] | 97/98 | 154/17.8 | anthranilate 1,2-dioxygenase small subunit  (EC 1.14.12.1) |  |  |
| ***ORF1*** | 735 | 1522474:1523208  reverse | 60 | ABC transporter, periplasmic substrate-binding protein  [*Pseudomonas* sp. VI4.1] | 89/93 | 244/26.7 | ABC transporter, periplasmic solute-binding protein subunit  (TCDB no. 3.A.1.4) | Benzoate/benzoate derivatives transport | |
| ***ORF2*** | 759 | 1523208:1523966  reverse | 62 | ABC transporter permease  [*Pseudoxanthomonas spadix*] | 85/94 | 252/27.3 | ABC transporter, periplasmic solute-binding protein subunit  (TCDB no. 3.A.1.4) |  |  |
| ***ORF3*** | 1044 | 1523963:1525006  reverse | 58 | ABC transporter permease  [*Pseudoxanthomonas spadix*] | 91/97 | 347/37.5 | ABC transporter permease subunit  (TCDB no.3.A.1.4) |  |  |
| ***ORF4*** | 861 | 1525017:1525877  reverse | 56 | ABC transporter permease  [*Ps. alcaliphila*] | 100/100 | 286/30.8 | ABC transporter permease subunit  (TCDB no. 3.A.1.4) |  |  |
| ***ORF5*** | 1119 | 1525937:1527055  reverse | 59 | ABC transporter ATP-binding protein [*Pseudoxanthomonas spadix*] | 95/97 | 37240.3 | ABC transporter ATP-binding protein subunit  (TCDB no. 3.A.1.4) |  |  |
| ***ORF6*** | 1182 | 1527231:1528412  reverse | 61 | ABC transporter ATP-binding protein [*Pseudoxanthomonas spadix*] | 96/98 | 393/42.0 | ABC transporter ATP-binding protein subunit  (TCDB no. 3.A.1.4) |  |  |
| ***benF*** | 1266 | 1528499:1529764  reverse | 59 | benzoate-specific porin [*Ps. saudiphocaensis*] | 89/94 | 421/46.7 | benzoate-specific porin  (TCDB no. 1.B.25) | Benzoate/benzoate derivatives uptake | |
| ***salJ*** | 192 | 1530036:1530227  reverse | 60 | 2-hydroxymuconate tautomerase  [*Ps. putida*] | 98/100 | 63/7 | 4-oxalocrotonate tautomerase (EC: 5.3.2.6) | Catechol degradation (*meta* cleavage pathway) | |
| ***salI*** | 795 | 1530276:1531070  reverse | 64 | 4-oxalocrotonate decarboxylase  [*Ps. stutzeri*] | 99/100 | 264/28.6 | 2-oxo-3-hexenedioate decarboxylase (EC: 4.1.1.77) |  |  |
| ***salH*** | 1041 | 1531067:1532107  reverse | 65 | 4-hydroxy-2-oxovalerate aldolase  [*Ps. stutzeri*] | 99/100 | 346/37.2 | 4-hydroxy-2-oxovalerate aldolase (EC: 4.1.3.39) |  |  |
| ***salG*** | 924 | 1532120:1533043  reverse | 65 | acetaldehyde dehydrogenase  [*Pseudomonas* spp.] | 99/99 | 307/33.0 | acetaldehyde dehydrogenase (acetylating) (EC: 1.2.1.10) |  |  |
| ***salF*** | 786 | 1533058:1533843  reverse | 65 | 2-oxopent-4-enoate hydratase  [*Ps. alcaliphila*] | 100/100 | 261/27.9 | 2-keto-4-pentenoate hydratase (EC: 4.2.1.80) |  |  |
| ***salE*** | 849 | 1533855:1534703  reverse | 67 | 2-hydroxymuconate-semialdehyde hydrolase  [*Ps. alcaliphila*] | 100/100 | 282/31.3 | 2-hydroxymuconate-semialdehyde hydrolase (EC: 3.7.1.9) |  |  |
| ***salD*** | 1461 | 1534714:1536174  reverse | 67 | 2-hydroxymuconate-6-semialdehyde dehydrogenase [*Ps. alcaliphila*] | 100/100 | 486/51.7 | 2-hydroxymuconate-6-semialdehyde dehydrogenase (EC: 1.2.1.85) |  |  |
| ***salC*** | 924 | 1536209:1537132  reverse | 58 | catechol 2,3-dioxygenase  [*Pseudomonas* spp.] | 100/100 | 307/35.0 | catechol 2,3-dioxygenase (EC: 1.13.11.2) |  |  |
| ***fdx*** | 339 | 1537129:1537467  reverse | 58 | chloroplast-type ferredoxin  [*Pseudomonas* spp.] | 100/100 | 112/12.5 | chloroplast-type ferredoxin | Salicylate hydroxylation | |
| ***salA*** | 1314 | 1537909:1539222  reverse | 59 | salicylate 1-monooxygenase  [*Ps. putida*] | 99/99 | 437/47.9 | salicylate 1-monooxygenase (EC: 1.14.13.1) |  |  |
| ***salR*** | 903 | 1539377:1540279  reverse | 55 | LysR family transcriptional regulator [*Pseudomonas* spp.] | 99/98 | 300/33.9 | LysR-family transcriptional activator | Regulation of transcription | |
| ***tbuX*** | 1374 | 1545237:1546610  reverse | 55 | aromatic hydrocarbon degradation membrane protein [*Ps.* *stutzeri*] | 99/99 | 457/48.2 | Aromatic hydrocarbon uptake transporter  (TCDB 1.B.9.2) | Aromatic hydrocarbons uptake | |
| ***bphD*** | 861 | 1546713:1547573  reverse | 56 | 2-hydroxy-6-oxo-6-phenylhexa-2,4-dienoate hydrolase [*Ps.* *pseudoalcaligenes* KF707] | 100/100 | 286/32.0 | 2-hydroxy-6-oxo-6-phenylhexa-2,4- dienoate hydrolase  (EC:3.7.1.8) | Benzoate/benzoate derivatives degradation | |
| ***bphI*** | 1041 | 1547644:1548684 reverse | 64 | 4-hydroxy-2-oxovalerate aldolase [*Ps.* *pseudoalcaligenes* KF707] | 100/100 | 346/36.8 | 4-hydroxy 2-oxovalerate aldolase (EC:4.1.3.39) | Catechol/catechol derivatives derivatives degradation (*meta* cleavage pathway) | |
| ***bphJ*** | 915 | 1548704:1549618  reverse | 64 | acetaldehyde dehydrogenase (acylating) [*Ps.* *pseudoalcaligenes* KF707] | 100/100 | 304/32.2 | acetaldehyde dehydrogenase (acylating) (EC:1.2.1.10) |  |  |
| ***bphH*** | 783 | 1549640:1550422  reverse | 65 | 2-hydroxypenta-2,4-dienoate hydratase (BphH) [Paraburkholderia xenovorans LB400] | 100/100 | 260/27.3 | 2-hydroxypenta-2,4-dienoate hydratase |  |  |
| ***bphK*** | 612 | 1550505:1551116  reverse | 61 | Glutathione S-transferase [*Ps.* *pseudoalcaligenes* KF707] | 100/100 | 203/22.4 | glutathione S-transferase | Biphenyl/PCB degradation | |
| ***bphC*** | 897 | 1551148:1552044  reverse | 60 | 2,3-dihydroxy-biphenyl 1,2-dioxygenase [*Ps.* *pseudoalcaligenes* KF707] | 100/100 | 298/32.5 | 2,3-dihydroxybiphenyl-1,2-dioxygenase  (EC 1.13.11.39) |  |  |
| ***bphB*** | 834 | 1552066:1552899  reverse | 61 | cis-2,3-dihydrobiphenyl-2,3-diol dehydrogenase [*Pseudomonas* spp.] | 100/100 | 277/28.9 | cis-2,3-dihydrobiphenyl-2,3-diol dehydrogenase  (EC:1.3.1.56) |  |  |
| ***bphG*** | 1227 | 1552946:1554172  reverse | 69 | biphenyl dioxygenase system ferredoxin-NAD reductase component [*Ps.* *pseudoalcaligenes* KF707] | 100/100 | 408/43.0 | biphenyl 2,3-dioxygenase ferredoxin reductase subunit  (EC:1.18.1.3) |  |  |
| ***bphF*** | 330 | 1554169:1554498  reverse | 56 | biphenyl dioxygenase system ferredoxin component [*Ps.* *pseudoalcaligenes* KF707] | 100/100 | 109/12.0 | biphenyl 2,3-dioxygenase ferredoxin subunit |  |  |
| ***ORF7*** | 420 | 1554573:1554992  reverse | 58 | BphX family protein [Alcanivorax sp. HA03] | 88/91 | 139/15.4 | biphenyl pathway membrane protein | Uknown | |
| ***bphE*** | 642 | 1555030:1555671  reverse | 58 | Biphenyl 2,3-dioxygenase small subunit [*Ps.* *pseudoalcaligenes* KF707] | 100/100 | 213/25.0 | biphenyl 2,3-dioxygenase small subunit  (EC 1.14.12.18) | Biphenyl/PCB degradation (terminal dioxygenase) | |
| ***bphA*** | 1377 | 1555714:1557090  reverse | 62 | Biphenyl 2,3-dioxygenase large subunit [*Ps.* *pseudoalcaligenes*] | 100/100 | 458/51.4 | biphenyl 2,3-dioxygenase large subunit  (EC 1.14.12.18) |  |  |
| ***bphR*** | 708 | 1557182:1557889  reverse | 58 | GntR-family transcriptional regulator [*Ps.* *pseudoalcaligenes* KF707] | 100/100 | 235/26.6 | GntR-family transcriptional regulator | Regulation of transcription | |
|  |  |  |  |  |  |  |  |  | |
| ***dmpI*** | 192 | 4397470:4397661  reverse | 60 | 2-hydroxymuconate tautomerase [*Pseudomonas* spp.] | 100/100 | 63/7.2 | 2-hydroxymuconate tautomerase  (EC 5.3.2.6) | Catechol/catechol derivatives degradation (*meta* cleavage pathway) | |
| ***dmpH*** | 795 | 4397716:4398510  reverse | 65 | 4-oxalocrotonate decarboxylase [*Ps.* *pseudoalcaligenes*] | 99/100 | 264/28.3 | 4-oxalocrotonate decarboxylase  (EC 4.1.1.77) |  |  |
| ***dmpG*** | 1035 | 4398510:4399544  reverse | 66 | 4-hydroxy-2-oxovalerate aldolase  [*Ps.* *pseudoalcaligenes*] | 100/100 | 344/37.3 | 4-hydroxy-2-oxovalerate aldolase  (EC 4.1.3.39 ) |  |  |
| ***dmpF*** | 939 | 4399556:4400494  reverse | 66 | Acetaldehyde dehydrogenase (acylating)  [*Ps.* *pseudoalcaligenes*] | 99/100 | 312/32.7 | Acetaldehyde dehydrogenase (acylating)  (EC 1.2.1.10) |  |  |
| ***dmpE*** | 786 | 4400510:4401295  reverse | 66 | 2-oxopent-4-enoate hydratase  [*Pseudomonas* spp.] | 100/100 | 261/27.8 | 2-oxopent-4-enoate hydratase  (EC 4.2.1.80) |  |  |
| ***dmpD*** | 849 | 4401307:4402155  reverse | 69 | 2-hydroxymuconic semialdehyde hydrolase  [*Pseudomonas* spp.] | 100/100 | 282/31.3 | 2-hydroxymuconic semialdehyde hydrolase  (EC 3.7.1.9) |  |  |
| ***dmpC*** | 1461 | 4402163:4403623  reverse | 66 | 2-hydroxymuconic semialdehyde dehydrogenase [*Pseudomonas* spp.] | 100/100 | 486/51.7 | 2-hydroxymuconic semialdehyde dehydrogenase  (EC 1.2.1.32) |  |  |
| ***dmpB*** | 924 | 4403660:4404583  reverse | 62 | catechol 2,3-dioxygenase [*Pseudomonas* spp.] | 100/100 | 307/35.2 | catechol 2,3-dioxygenase  (EC 1.13.11.2) |  |  |
| ***fdx*** | 339 | 4404580:4404918  reverse | 65 | chloroplast-type ferredoxin  [*Ps.* *pseudoalcaligenes*] | 99/100 | 112/12.2 | chloroplast-type ferredoxin | Phenol/phenolics hydroxylation | |
| ***pheP*** | 1062 | 4404927:4405988  reverse | 65 | phenol 2-monooxygenase P5 component  [*Pseudomonas* sp. CF600] | 99/100 | 353/38.5 | Phenol 2-monooxygenase subunit P5  (EC 1.14.13.7) |  |  |
| ***pheO*** | 360 | 4405999:4406358  reverse | 61 | Phenol 2-monooxygenase P4 component  [*Pseudomonas* sp. CF600] | 100/100 | 119/13.2 | Phenol 2-monooxygenase subunit P4  (EC 1.14.13.7) |  |  |
| ***pheN*** | 1554 | 4406426:4407979  reverse | 60 | Phenol 2-monooxygenase P3 component  [*Ps. putida*] | 99/99 | 517/60.6 | Phenol 2-monooxygenase P3 subunit  (EC 1.14.13.7) |  |  |
| ***pheM*** | 273 | 4407991:4408263  reverse | 60 | Phenol 2-monooxygenase P2 component  [*Pseudomonas* sp. gl06]] | 100/100 | 90/10.5 | Phenol 2-monooxygenase P2 subunit  (EC 1.14.13.7) |  |  |
| ***pheL*** | 996 | 4408267:4409262  reverse | 63 | Phenol 2-monooxygenase P1 component  [*Ps. pseudoalcaligenes*] | 100/100 | 331/38.2 | Phenol 2-monooxygenase P1 subunit  (EC 1.14.13.7) |  |  |
| ***pheK*** | 270 | 4409315:4409584  reverse | 60 | Phenol 2-monooxygenase P0 component  [*Pseudomonas* sp. CF600] | 100/100 | 89/10.3 | Phenol 2-monooxygenase P0 subunit  (EC 1.14.13.7) |  |  |
| ***dmpR*** | 1692 | 4410002:4411693  forward | 64 | sigma-54-dependent Fis family transcriptional regulator [*Ps. chengduensis*] | 98/98 | 563/63.3 | Fis-family transcriptional regulator | Regulation of transcription | |
|  |  |  |  |  |  |  |  |  |  |
| ***pcaC*** | 396 | 4445675:4446070  reverse | 63 | 4-carboxymuconolactone decarboxylase  [*Ps. sihuiensis*] | 100/100 | 131/14.9 | 4-carboxymuconolactone decarboxylase  (EC 4.1.1.44) | Protochatechuate degradation  *(ortho* cleavage pathway) |  |
| ***pcaD*** | 789 | 4446169:4446957  reverse | 66 | 3-oxoadipate enol-lactonase  [*Ps. alcaliphila*] | 99/99 | 262/28.3 | 3-oxoadipate enol-lactonase  (EC 3.1.1.24) |  |  |
| ***pcaB*** | 1353 | 4446967:4448319  reverse | 72 | 3-carboxy-*cis,cis*-muconate cycloisomerase [*Ps. alcaliphila*] | 96/97 | 450/47.7 | 3-carboxy-cis,cis-muconate cycloisomerase  (EC 5.5.1.2) |  |  |
| ***pcaF*** | 1206 | 4448601:4449806  reverse | 67 | 3-oxoadipyl-CoA thiolase  [*Ps. sihuiensis*] | 99/99 | 401/42.0 | 3-oxoadipyl-CoA thiolase  (EC 2.3.1.16) |  |  |
| ***pcaJ*** | 783 | 4449803:4450585  reverse | 68 | 3-oxoadipate--succinyl-CoA transferase [*Ps. oleovorans/pseudoalcaligenes group*] | 99/100 | 260/27.2 | 3-oxoadipate--succinyl-CoA transferase subunit  (EC 2.8.3.6) |  |  |
| ***pcaI*** | 849 | 4450585:4451433  reverse | 67 | 3-oxoadipate:succinyl-CoA transferase subunit A  [*Ps. toyotomiensis*] | 99/100 | 282/30.8 | 3-oxoadipate:succinyl-CoA transferase subunit  (EC 2.8.3.6) |  |  |
| ***mcpS*** | 870 | 4451559:4452431 reverse | 75 | Methyl-accepting chemotaxis protein McpS [*Ps. oleovorans* subsp. *oleovorans*] | 99/99 | 289/30.0 | Methyl-accepting chemotaxis protein | chemotaxis |  |
| ***ORF8*** | 1281 | 4452481:4453761  reverse | 68 | porin (OprD family)  [*Ps. mendocina*] | 98/99 | 426/46.1 | outer membrane porin OprD family (small molecules transport) | unknown |  |
| ***benK*** | 1209 | 4453847:4455055  reverse | 71 | benzoate transporter [*Ps. aeruginosa*] | 99/99 | 402/41.2 | Benzoate:H^+^ Symporter (BenE) Family  (TCDB no. 2.A.46) | Benzoate/benzoate derivatives uptake |  |
| ***catA*** | 942 | 4455177:4456118  reverse | 68 | catechol 1,2-dioxygenase [*Ps. oleovorans* subsp. *oleovorans*] | 99/100 | 313/34.6 | catechol 1,2-dioxygenase (EC 1.13.11.1) | Catechol degradation  (*ortho* cleavage pathway) |  |
| ***catC*** | 291 | 4456149:4456439  reverse | 67 | muconolactone delta-isomerase  [*Pseudomonas* sp. Leaf83] | 100/100 | 96/11.1 | muconolactone delta-isomerase  (EC 5.3.3.4) |  |  |
| ***catB*** | 1122 | 4456456:4457577  reverse | 69 | muconate cycloisomerase  [*Ps. pseudoalcaligenes*] | 99/100 | 373/39.9 | muconate cycloisomerase  (EC 5.5.1.1) |  |  |
| ***pcaK*** | 1341 | 4457613:4458953  reverse | 68 | major facilitator transporter  [*Ps. pseudoalcaligenes*] | 100/100 | 446/47.1 | Putative benzoate transport protein (MFS superfamily)  (TCDB no. 2.A.1) | Benzoate/benzoate derivatives uptake |  |
| ***benD*** | 765 | 4459071:4459835  reverse | 70 | 1,6-dihydroxycyclohexa-2,4-diene-1-carboxylate dehydrogenase  [*Ps. oleovorans*] | 99/99 | 254/27.1 | 1,6-dihydroxycyclohexa-2,4-diene-1-carboxylate dehydrogenase  (EC 1.3.1.25) | Benzoate/benzoate derivatives degradation |  |
| ***benC*** | 1011 | 4460046:4461056  reverse | 66 | benzoate/toluate 1,2-dioxygenase reductase subunit  [*Ps. aeruginosa*] | 99/99 | 336/36.1 | benzoate/toluate 1,2-dioxygenase reductase subunit  (EC 1.14.12.10) | Benzoate/benzoate derivatives degradation (terminal dioxygenase) |  |
| ***benB*** | 489 | 4461139:4461627  reverse | 61 | benzoate 1,2-dioxygenase small subunit  [*Pseudomonas* sp. Leaf83] | 99/100 | 162/19.4 | benzoate 1,2-dioxygenase small subunit  (EC 1.14.12.10) |  |  |
| ***benA*** | 1362 | 4461628:4462989  reverse | 64 | benzoate 1,2-dioxygenase large subunit  [*Ps. aeruginosa*] | 99/100 | 453/51.8 | benzoate 1,2-dioxygenase large subunit  (EC 1.14.12.10) |  |  |
| ***benR*** | 957 | 4463284:4464240  reverse | 64 | AraC family transcriptional regulator  [*Ps. oleovorans*] | 99/100 | 318/35.9 | *ben* operon transcriptional activator | Regulation of transcription |  |
| ***pcaR*** | 846 | 4464586:4465431  reverse | 68 | IclR family transcriptional regulator [*Pseudomonas* sp. 21C1] | 97/98 | 281/31.2 | transcriptional regulator |  |  |
| ***ORF9*** | 948 | 4465593:4466540  reverse | 66 | putative transporter YfdV  [*Ps. oleovorans* subsp. *oleovorans*] | 99/100 | 315/32.9 | Permease  (TCDB no. 2.A.69) | unknown |  |
| ***zapE*** | 1011 | 4466534:4467544  reverse | 66 | cell division protein ZapE  [*Ps. alcaliphila*] | 99/100 | 336/38.2 | cell division protein | unknown |  |
| ***pcaG*** | 609 | 4467546:4468154  reverse | 65 | protocatechuate 3,4-dioxygenase subunit alpha [*Pseudomonas* sp. Leaf83] | 99/100 | 202/22.3 | protocatechuate 3,4-dioxygenase α subunit  (EC 1.13.11.3) | Protochatechuate (*ortho* cleavage pathway) (terminal dioxygenase) |  |
| ***pcaH*** | 720 | 4468166:4468885  reverse | 63 | protocatechuate 3,4-dioxygenase subunit beta [*Ps. pseudoalcaligenes*] | 99/100 | 239/27.1 | protocatechuate 3,4-dioxygenase β subunit  (EC 1.13.11.3) |  |  |
| ***pcaQ*** | 933 | 4469002:4469934  reverse | 68 | *pca* operon transcription factor PcaQ  [*Pseudomonas* sp. Leaf83] | 100/100 | 310/33.3 | transcription factor | Regulation of transcription |  |

^*)^ indicates position in the JAB1-strain annotated genome (GenBank accession no. NZ_CP016162.1)

^**)^ Searches through Non-redundant (nr) and Reference proteins (refseq_protein), UniProtKB/Swiss-Prot (swissprot) databases and, in case of putative transmembrane transporters, The Transporter Classification Database (TCDB [1]) were used for assessment of putative functions of respective gene products. The identity/similarity values of the top relevant hit from nr database are indicated.

^***)^ Molar weight was assessed by SnapGene software (GSL Biotech).

Reference:

1. Saier MH, Jr., Reddy VS, Tsu BV, Ahmed MS, Li C et al. The Transporter Classification Database (TCDB): recent advances. Nucleic Acids Res 2016;44(D1):D372-379.
